# Comparative Genomic Analysis of 19 Clinical Isolates of Tigecycline-Resistant *Acinetobacter baumannii*

**DOI:** 10.3389/fmicb.2020.01321

**Published:** 2020-07-07

**Authors:** Lin Liu, Ping Shen, Beiwen Zheng, Wei Yu, Jinru Ji, Yonghong Xiao

**Affiliations:** ^1^State Key Laboratory for Diagnosis and Treatment of Infectious Diseases, The First Affiliated Hospital, College of Medicine, Zhejiang University, Hangzhou, China; ^2^Collaborative Innovation Center for Diagnosis and Treatment of Infectious Diseases, Zhejiang University, Hangzhou, China

**Keywords:** *Acinetobacter baumannii*, tigecycline resistance, comparative genomics, antibiotic resistance genes, virulence factors, nosocomial pathogen

## Abstract

To assess the genomic profiles of tigecycline (Tgc)-resistant *Acinetobacter baumannii*, including antibiotic resistance (AR) genes and virulence factors (VF), whole-genome shotgun sequencing was performed on 19 Tgc-resistant (TgcR) *A. baumannii* strains collected in a tertiary hospital during the early phase of the clinical introduction of Tgc in China from late 2012 to mid-2014. The major sample types containing TgcR strains were sputum and drain fluid. Data from an average of 624 Mbp of sequence was generated on each bacterial genome, with Q30 quality of 90%, and an average coverage of 96.6%. TCDC-AB0715 was used as a reference genome. The genome sequences were annotated for functional elements including AR genes, VFs, genome islands, and inserted sequences before they were comparatively analyzed. The antibiotic susceptibility phenotypes of the strains were examined by a broth microdilution method to determine the minimal inhibitory concentration (MIC) of strains against major clinical antibiotics. The AR genes (ARGs) were annotated using the Comprehensive Antibiotic Resistance Database (CARD). Thirty-three ARGs were shared by all 19 TgcR strains, and 24 ARGs were distributed differently among strains. A total of 391 VFs were found to be diversely distributed in all TgcR strains. Based on ARG number distribution, the 19 TgcR strains were divided into several groups. Highly differentiated genes included *gpi*, *mphG*, *armA*, *msrE*, *adec*, *catB8*, *aadA*, *sul1*, *bla*_OXA–__435_, *aph3i*, and *bla*_TEM–__1_, which may represent gene markers for TgcR *A. baumannii* sub-types. In addition, when compared with Tgc-sensitive (TgcS) strains collected during the same period, TgcR strains featured enrichment of ARGs including *aph6id*, *aph3ib*, and *teta*. Compared with 26 other whole-genome sequences of *A. baumannii* deposited in GeneBank, TgcR strains in this study commonly lacked the EF-Tu mutation for elfamycin resistance. Previous investigation of three *A. baumannii* strains isolated from one patient indicated genomic exchange and a homologous recombination event associated with generation of tigecycline resistance. This study further analyzed additional TgcR strains. Phylogenetic analysis revealed a close evolutionary relationship between 19 TgcR strains and to isolates in East and Northeast China. In short, the comprehensive functional and comparative genomic analysis of 19 clinical TgcR *A. baumannii* strains isolated in the early stage of Tgc usage in China revealed their close phylogenetic relationship yet variable genetic background involving multiple resistance mechanisms. Using a simple ARG or VF gene number diversity method and marker genes, TgcR strain sub-types can be identified. The distinct characteristics of TgcR *A. baumannii* strains with versatile genomic resistance and regulation patterns raise concern regarding prediction and control of Tgc resistance in the clinic.

## Introduction

*Acinetobacter baumannii* is a nosocomial pathogen that is often detected in intensive care settings (Li et al., 2015). Infections caused by this pathogen include ventilator-associated pneumonia, bloodstream infections, burn infections, meningitis, soft tissue infections, osteomyelitis, and endocarditis (Perez et al., 2007; McConnell et al., 2013). The rapid rise of multidrug resistance (MDR) in *A. baumannii* strains has raised serious concerns about the diminishing arsenal of effective antimicrobial agents (Fischbach and Walsh, 2009). In addition to antibiotics, *A*. *baumannii* can resist various types of environmental stress, such as desiccation, detergents, and radioactivity, which contributes to its wide prevalence (Zhang et al., 2013; Pomba et al., 2014; Brahmi et al., 2016) and transmission (Kempf and Rolain, 2012). The global challenges of MDR *A*. *baumannii* demands a better understanding of the mechanisms contributing to its robust capacity for antibiotic resistance (AR).

Tigecycline (Tgc) is a novel 9-t-butyl-substituted minocycline derivative and is effective against a broad spectrum of Gram-positive and Gram-negative pathogens, a significant proportion of which are resistant to many currently available antibiotics (Stein and Babinchak, 2013). In recent years, however, emergence of Tgc-resistant (TgcR) *A*. *baumannii* strains has been reported (Costello et al., 2015). The ability to acquire multidrug resistance may occur via different mechanisms, i.e., horizontal gene transfer carrying resistance to several drugs or increased production of efflux systems that can remove antimicrobial compounds (Roca et al., 2012; Blair et al., 2015). Previous studies have revealed that Tgc resistance in *A*. *baumannii* is associated with the overexpression of resistance-nodulation-division (RND) efflux pumps, such as *adeABC* (Magnet et al., 2001; Ruzin et al., 2010), *adeIJK* (Damier-Piolle et al., 2008), and *adeFGH* (Coyne et al., 2010). Expression of *adeABC* is tightly regulated by the *adeRS* two-component system (Marchand et al., 2004). In addition, a mutation in *trm*, a tigecycline-related-methyltransferase gene, can reduce Tgc levels in *A*. *baumannii* (Chen et al., 2014). The gene encoding TetX1, a flavin-dependent monooxygenase that can modify tetracyclines and Tgc, has been detected in cases with reduced Tgc sensitivity (Deng et al., 2014).

Tigecycline has been approved for clinical use in many countries, including the United States in 2007 and China in late 2012. This was quickly followed by the emergence of TgcR *A. baumannii* isolates (Zhu et al., 2013; Liu et al., 2016). To investigate the molecular mechanisms underlying this trend, 19 clinical TgcR *A*. *baumannii* isolates were collected in a tertiary hospital in Eastern China between late 2012 and mid-2014 and were subjected to epidemiological, genomic, and expression analyses. Based on similarities in ARG or VF gene number distribution, the 19 TgcR strains were separable into four groups. Our results illuminate key components conferring the spontaneous rise of Tgc resistance in a hospital environment.

## Materials and Methods

### Isolation and Susceptibility Profile Characterization

Nineteen *A. baumannii* strains were collected from the First Affiliated Hospital, Zhejiang University School of Medicine, from 2012 to 2014. Eight strains were collected from three different patients at different stages. The MICs of several antibiotics were determined for the isolated strains using broth microdiluton methods. Related clinical data including sample type, time of collection, ward, patient information (age, sex, and antibiotic usage) were retrieved and analyzed.

### Genomic Sequencing and Assembly

DNA was isolated using a QiaAmp DNA Mini kit. Paired-end libraries were constructed using a NEBNext DNA library preparation kit. Each library was sequenced by Wuxi AppTec using a HiSeq^TM^ 2000 (Illumina, United States) in the paired-end mode with a read length of 100 bp. The Illumina sequence data was assembled using the SOAPdenovo (version 2.04) package (Liu et al., 2016). The total number of contigs, genome size, G + C content, and total coverage was obtained.

### Genome Annotation

The assembled genome sequence was annotated using the NCBI Prokaryotic Genome Automatic Annotation Pipeline (PGAAP). Glimmer software (version 3.0) was used for the identification of protein-coding genes (Delcher et al., 2007), tRNAscan-SE for the identification of tRNA genes (Lowe and Eddy, 1997), and RNAmmer for the identification of rRNA genes (Lagesen et al., 2007). Insertion sequences (ISs) were identified using the IS Finder database^1^ (Siguier et al., 2006). The databases of ARDB and CARD were used for the drug resistance gene annotation. Amino acid sequences of predicted genes were aligned against the proteins in these databases using blastp. A gene was assigned to an AR protein by the highest score hit containing a minimum identity of 40%. The annotation of virulence gene was performed using the VFDB database by blastp (parameters: “-e 1e-5-F F”).

### Comparison of Analyzed Genomes

Core-pan genome analysis was used to estimate the number of shared genes (core genes) and unique genes among the isolated strains using the PanOCT program (Fouts et al., 2012). Data used in comparative analysis were downloaded from the NCBI database^2^, including complete sequences and annotation of *A. baumannii* isolates. Genome level differences between different isolates were analyzed. SNPs were identified with SNPFinder and Mauve (Darling et al., 2004; Song et al., 2005). IS sequences were detected with IslandViewer (Langille and Brinkman, 2009). The phylogenic tree was constructed on the basis of core genome SNPs using TreeBestV1.9.2.

### Nucleotide Sequence Accession Numbers

The data from this whole-genome shotgun project have been deposited at GenBank.

### Ethics Statement

No animal studies are presented in this manuscript. No human studies are presented in this manuscript. No potentially identifiable human images or data is presented in this study.

## Results

### Strain Types, AR Profiles, and Clinical Information

From late 2012 to mid-2014, 19 TgcR (MIC ≥ 4) and 37 Tgc-intermediate (2 ≤ MIC < 4) strains were isolated using a broth microdilution method in a tertiary hospital in Eastern China. Three TgcR strains were previously reported with TgcR-related genomic mutation and transcriptomic regulation (Liu et al., 2016). In this report, we sequenced the additional 17 TgcR strains, thereby comparatively analyzing molecular traits related to Tgc resistance in *A. baumannii*.

Molecular epidemiological analysis showed that ST195 (52, 57.8%) was the most prevalent type, followed by ST208 (8, 8.9%), ST191 (6, 6.7%), and ST784 (6, 6.7%) (Supplementary Table S1). Other sequence types (STs) included ST642, ST643, ST451, ST547, ST368, and ST369. Sputum and drain fluid were the most common sample types from which TgcR strains were isolated. ICUs were the most endemic sites where TgcR strains were isolated. In a comparison of 20 non-repetitive Tgc-sensitive (TgcS) strains and antibiotic usage, patients from whom TgcR strains were isolated received more antibiotic treatment with quinolone, cefoperazone/sulbactam, or piperacillin/tazobactam (Supplementary Table S2). Moreover, a quarter of the patients from whom the TgcR strains were isolated received tigecyline treatment, and none of the patients from whom TgcS strains were isolated had received tigecycline. It should be noted that TgcR strains were not necessarily isolated during or after usage of tigecycline.

The minimal inhibitory concentrations (MICs) against major clinical antibiotics were measured for 19 TgcR strains (Table 1). All strains were resistant to at least three categories of antibiotics, and only susceptible or intermediately resistant to amikacin and levofloxacin.

**TABLE 1:** Antibiotic resistance profiles (MIC, μg/ml) of 19 TgcR *Acinetobacter baumannii* strains against major clinical antibiotics.

	2015ZJAB2	2015ZJAB3	2015ZJAB9	2015ZJAB10	2015ZJAB11	2015ZJAB12	2015ZJAB13	2015ZJAB14	2015ZJAB15	2015ZJAB16
Cefepime	256	64	≥128	128	≥128	≥128	≥128	≥128	128	128
Cefoperazone/sulbactam	32	64	128	64	128	64	128	128	64	128
Ceftazidime	32	32	128	≥128	≥128	≥128	≥128	≥128	128	128
Levofloxacin	128	128	16	8	8	4	32	16	16	16
Imipenem	2	2	64	64	128	64	32	64	32	64
Colistin	32	16	4	8	8	8	16	16	16	16
Tigecycline*	8	6	4	4	4	4	4	4	4	4

	**2015ZJAB17**	**2015ZJAB18**	**2015ZJAB19**	**2015ZJAB20**	**2015ZJAB21**	**2015ZJAB22**	**2015ZJAB23**	**2015ZJAB24**	**2015ZJAB25**	

Cefepime	≥128	≥128	128	≥128	≥128	128	128	64	≥128	
Cefoperazone/sulbactam	64	128	64	≥128	64	128	≥128	64	128	
Ceftazidime	≥128	≥128	≥128	≥128	≥128	≥128	128	64	≥128	
Levofloxacin	32	32	16	16	≥32	8	≥32	16	≥32	
Imipenem	64	64	64	128	64	64	≥128	32	64	
Colistin	32	32	32	8	8	16	2	16	8	
Tigecycline*	4	4	4	4	4	4	4	4	4	

*MIC was measured by broth microdilution methods following CLSI M07-A9 guidelines. ^∗^Tigecycline susceptibility was interpreted according to EUCAST 2016 v6.0.*

### Sequencing and Data Assembly

Sequencing data and genome assembly results are shown in Supplementary Table S3. High-quality data from an average of 624.45 Mbp of sequence was generated for the 19 strains, corresponding to an average sequencing depth of approximately 156-fold. An average of 3,759 coding DNA sequences (CDSs) was predicted. The average GC content of isolated *A. baumannii* strains was 38.86%.

### Comparative Genomic Analysis and Phylogenetic Tree

The 19 TgcR *A. baumannii* genomes were functionally annotated for ARGs, VFs, mobile elements including ISs and Genomic Islands (GIs; detailed later) and common traits. Unique characteristics of strains were compared and analyzed.

**FIGURE 1:**
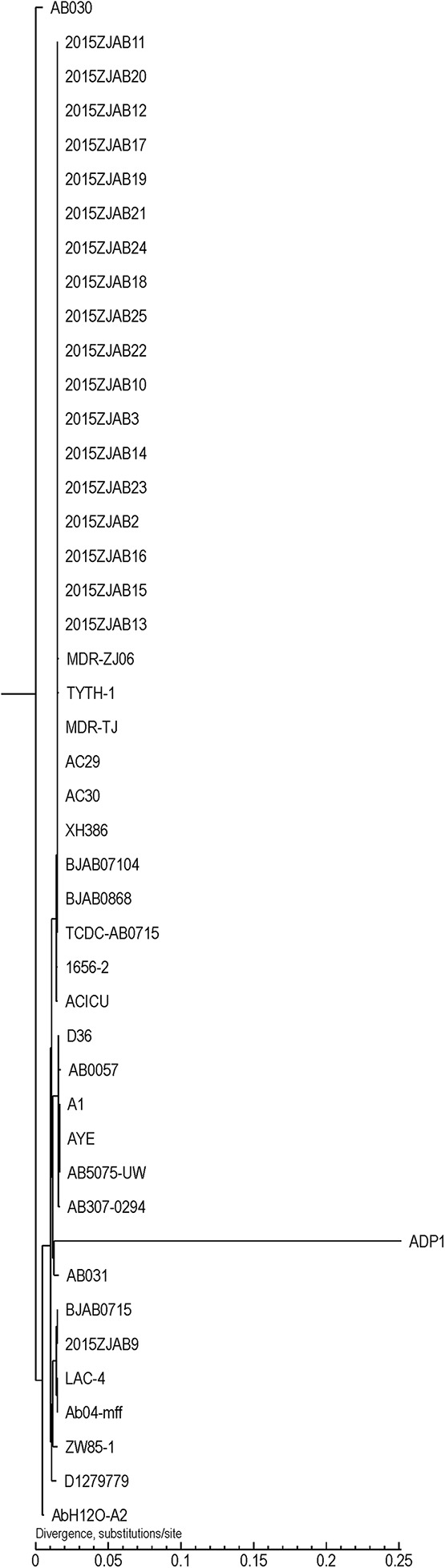
Phylogenetic tree of 19 tigecycline-resistant (TgcR) strains involved in this study and *Acinetobacter baumannii* strains retrieved from GeneBank based on core gene SNPs.

Using core gene-based TreeBestV1.9.2 software, a phylogenetic tree was constructed for *A. baumannii* strains (Figure 1). TCDC-AB0715 was used as a reference because it has the highest coverage among studied genomes. The 19 TgcR strains were phylogenetically similar to strains isolated in East and Northeast China. However, strains isolated from the same patient were not necessarily in the same subclade.

#### Colinearity Analysis and Intra-Group Comparison of SNPs and InDels

Strains isolated from the same patient at different time points were grouped together and subjected for intra-group comparative studies to reveal genomic relatedness. Among the 17 new TgcR strains, 6 were isolated from three patients (three groups). The first and earliest-isolated strain was considered the intra-group reference for detection of SNPs, InDels, and large structural variations by colinearity analysis within each group (patient).

In Group 1, 1,034 synonymous and 344 non-synonymous mutations were identified in strains 2015ZJAB12 and 2015ZJAB13 (Table 2). These SNPs were intensively clustered in three scaffolds, and spare regional variation occurred across the entire genome, raising the possibility of genomic region exchange. Specifically, stop mutations that may alter proteins’ functions included premature stop mutations in the MarC family membrane transporter gene *SnatA*, glycosyl hydrolase, two Vi polysaccharide biosynthesis proteins, VipA, TviB, heme-binding protein A, and a probable tape measure protein according to SwissProt annotation. Multiple genes discovered to be mutated were related to cell surface structure (cell wall and membrane) and virulence.

**TABLE 2:** Statistical analysis of intra-group SNPs.

Type	Patient 1	Patient 2		Patient 3
	2015ZJAB12 vs. 2015ZJAB13	2015ZJAB21 vs. 2015ZJAB22	2015ZJAB21 vs. 2015ZJAB23	2015ZJAB19 vs. 2015ZJAB20
Start_syn	0	0	0	0
Stop_syn	0	0	1	0
Start_nonsyn	0	0	4	0
Stop_nonsyn	5	1	7	2
Premature_stop	9	0	12	0
Synonymous	1034	1	3,772	6
Non-synonymous	344	6	981	8
Total_CDS_SNP	1389	8	4,773	13
Intergenic	242	2	591	18
Total_SNP	1631	10	5,364	31

Of the Group-2 strains, 2015ZJAB23 may derive from a different strain than 2015ZJAB22, indicating a possible re-infection. Strain 2015ZJAB23 contained four non-synonymous start mutations and seven non-synonymous stop mutations. Proteins with premature stop codons included stearoyl-CoA 9-desaturase electron transfer partner, UTP-glucose-1-phosphate uridylyltransferase, tyrosine-protein kinase PTK, Na(+)/H(+) ion antiporter NhaA, *trans-*acting regulatory protein HvrA, UPF0056 membrane protein BU449, outer membrane protein OprM, and probable tape measure protein OS.

**FIGURE 2:**
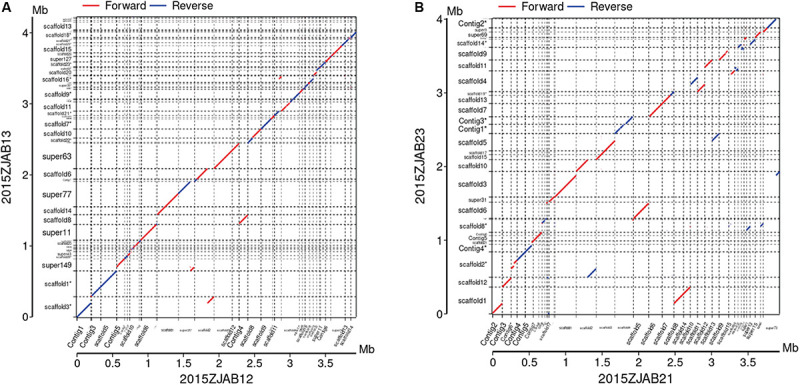
Colinearity analysis between 2015ZJAB12 and 2015ZJAB13 **(A)**, and 2015ZJAB21 and 2015ZJAB23 **(B)**.

Colinearity analysis revealed large structural changes of genomes as shown in Figure 2. In the group of strains isolated from Patient 3, there were few SNPs and a high colinearity relationship (Liu et al., 2016).

### Functional Annotation and Comparative Analysis

Functional CDS, ARGs, VFs, GIs, ISs, and integron elements were annotated and compared between TgcS and other MDR *A. baumannii* genomes. GO terms, COG categories, and KEGG pathways were analyzed to reveal metabolic characteristics.

#### Genome Islands, Plasmids, and Insertion Sequences

**FIGURE 3:**
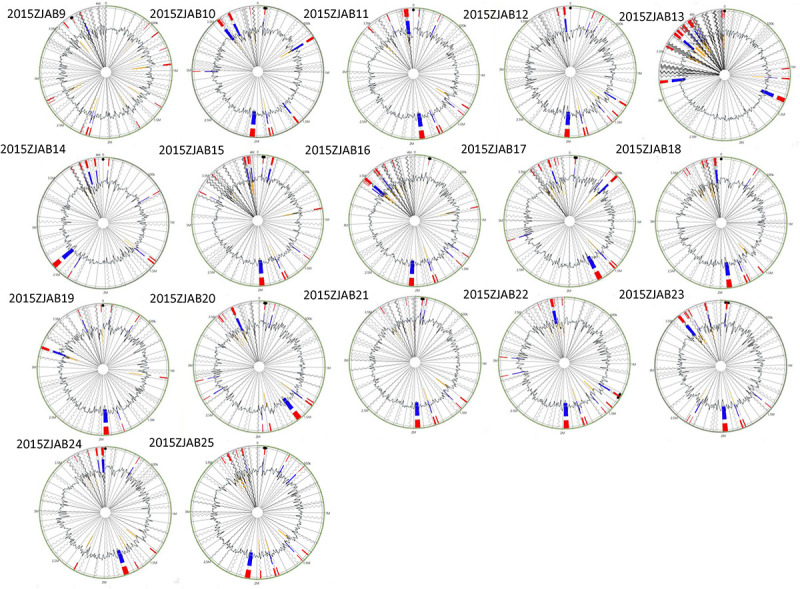
Gene island forecast in the genome of 17 TgcR strains. Red represents the predicted results after integration of the four methods. Blue represents the results predicted by the IslandPath-DIMOB method. Yellow represents the SIGH-HMM method.

The distribution of GIs in each TgcR strain is shown in Figure 3. An average of 13 GIs were present in the TgcR strains, with 2015ZJAB13 having the most (20) GIs, and 2015ZJAB19 and 2015ZJAB24 having the least (9) GIs. Additionally, multiple ARGs were associated with GIs. The most ARGs were found in *aph33ib*, *aph6id*, *aph3ia*, *bl2b_tem1*, and *sul1* genes, which are related to antimicrobial resistance to aminoglycoside, beta-lactam, and sulfonamide.

Using a query coverage of 70% as a cutoff, plasmids homologous to p2BAB07104, pMDR-ZJ06, p1ABIBUN, and pAB120 were revealed.

In total, 82 ISs belonging to seven families were identified in TgcR strains (Table 3). IS*Vsa3* (19/19) and IS*Aba1* (18/19) were the most prevalent IS elements. IS*Aba26*, IS*Aba33*, IS*26*, IS*15DI*, IS*15DII*, and IS*15DIV* were found in 17 out of 19 strains. The least common IS types were IS*Aha1*, IS*Aha2*, IS*Aha3*, IS*Aba5*, IS*Aba7*, IS*Aba12*, IS*Aba13*, IS*17*, IS*Ajo1*, and IS*Alw1*, which were only detected in strain 2015ZJAB13, indicating frequent genomic information exchange in its evolutionary history. In the phylogenetic tree, it is shown that 2015ZJAB13 was the most divergent strain from the other TgcR isolates, which was possibly related to frequent insertion of exogenous sequences.

**TABLE 3:** Statistical summary of insertion sequence (IS) elements in 19 TgcR *A. baumannii* strains.

Subject_annotation	No.	Subject_id	No.	Subject_id	No.
Family: IS256	17	ISVsa3	19	ISAha3	1
Family: IS3	18	ISAba1	18	ISAjo1	1
Family: IS4	19	IS15DI	17	ISAlw1	1
Family: IS5	15	IS15DII	17		
Family: IS6	17	IS15DIV	17		
Family: IS66	14	IS26	17		
Family: IS91	19	ISAba26	17		
		ISAba33	17		
		IS1006	16		
		ISAba21	15		
		ISAba22	15		
		ISEc28	15		
		ISEc29	15		
		ISAba24	14		
		ISEc35	4		
		ISAba18	2		
		ISAba19	2		
		ISAba2	2		
		ISAba29	2		
		ISAba34	2		
		IS17	1		
		ISAba12	1		
		ISAba13	1		
		ISAba20	1		
		ISAba5	1		
		ISAba7	1		
		ISAha1	1		
		ISAha2	1		

#### Annotation of AR Genes Using Antibiotic Resistance Gene Database (ARDB) and Comprehensive Antibiotic Resistance Database (CARD)

**FIGURE 4:**
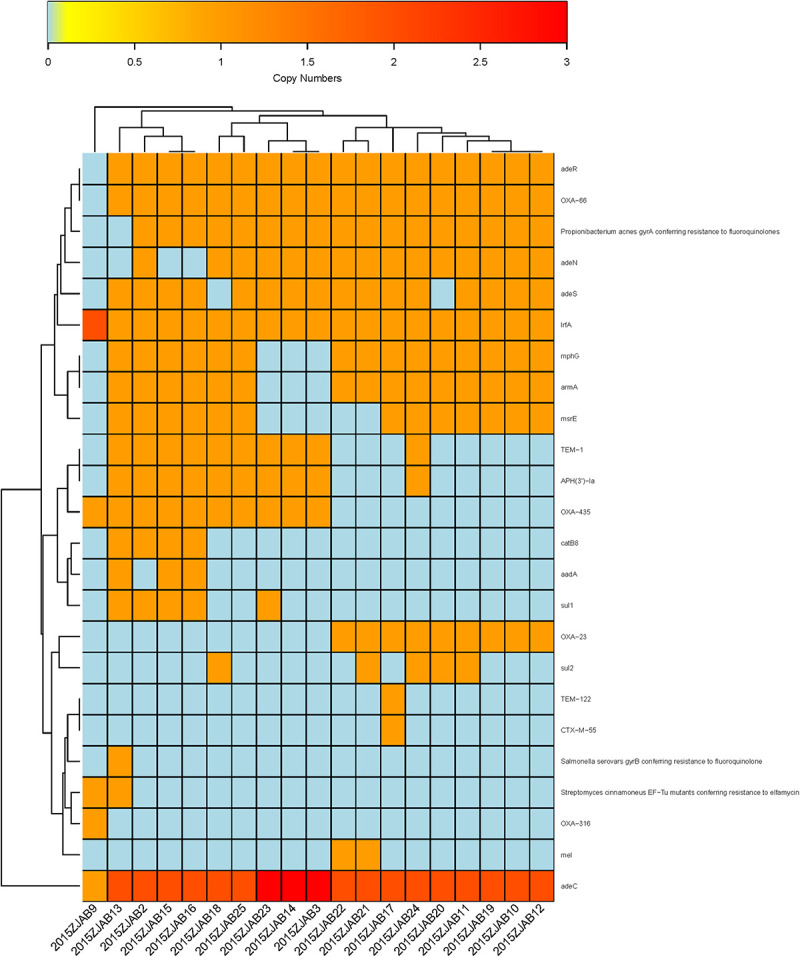
The heatmap of antibiotic resistance gene number distribution in the 19 TgcR strains.

Using ARDB, 31 ARGs were annotated in TgcR strains, and 33 ARGs were annotated by CARD. The latter database has been more frequently updated based on protein sequences. Therefore, this study mainly adopted CARD annotations. Based on gene number diversity, 24 ARGs were found to vary from zero to three copies in the TgcR strains (*bla*_OXA–__66_, *gyrA*, *adeR*, *adeS*, *mphG*, *armA*, *msrE*, *adeN*, *bla*_OXA–__23_, *lrfA*, *bla*_TEM–__1_, *aph3ia*, *bla*_OXA–__435_, *catB8*, *aadA*, *sul1*, *bla*_TEM–__122_, *bla*_CTX–M–__55_, *gyrB*, *EF-Tu mutant*, *bla*_OXA–__316_, *mel*, *sul2*, *and adeC*). Among these varied ARGs, *lrfA* and *adeC* were present in all strains, including 2015ZJAB9, but with different numbers. OXA-66 was only absent in 2015ZJAB9. *Propionibacterium acnes gyrA* (17/19), *adeR* (18/19), *adeS* (16/19), *mphG* (15/19), *armA* (15/19), and *adeN* (15/19) were also widely distributed among strains. *APH3’1a* and *OXA-435* genes were present in approximately half of the TgcR strains. The least common ARGs were *sul1* (5/19), *catb8* (4/19), and *aadA* (3/19). EF-Tu mutants (2/19) were only present in strains 2015ZJAB9 and 2015ZJAB13. Mutants of *mel* (2/19) were only present in strains 2015ZJAB21 and 2015ZJAB22. Those of *ctx-M-55* were only found in one strain, 2015ZJAB17. Thus, this simple gene number method can efficiently divide the strains into at least three sub-groups (Figure 4) differentiated by the presence of a few marker genes. It should be noted that strain 2015ZJAB9 had distinct ARG patterns.

The first sub-group contained strains 2015ZJAB22, 2015ZJAB21, 2015ZJAB17, 2015ZJAB24, 2015ZJAB20, 2015ZJAB11, 2015ZJAB19, 2015ZJAB10, and 2015ZJAB12 (Figure 4). The second sub-group included strains 2015ZJAB18, 2015ZJAB25, 2015ZJAB23, 2015ZJAB14, and 2015ZJAB3. The third sub-group contained strains 2015ZJAB13, 2015ZJAB2, 2015ZJAB15, and 2015ZJAB16. The most unique and abundant ARGs carried by the third sub-group were *catB8*, *aadA*, *gryB*, and *EF-Tu*. A lack of *mphG* and *armA* marked the second sub-group. *Mel*, *bla*_TEM–__122_, *ctx-M-55*, and *cxa-23* were only present in the first sub-group.

There were eight different resistance types (*bla*_TEM–__1_, *aph3ia*, *bla*_OXA–__435_, *catB8*, *aadA*, *sul1*, *gyrB*, and *EF-Tu mutant*) in the two strains of Patient 1, with strain 2015ZJAB13 having the highest number of ARGs. Between TgcR strains 2015ZJAB19 and 2015ZJAB20 isolated from Patient 3, only *sul2* number distribution was different, which was in accordance with a prior intra-group comparison showing high similarity between the two genomes. In strains isolated from Patient 2, only s*ul2* gene number was different between 2015ZJAB21 and 2015ZJAB22. ARGs were distributed differently in 2015ZJAB23. Among all isolates, 2015ZJAB9 had the lowest number of ARGs. With CARD annotation, the numbers of different ARGs in the three patients were 11, 9, and 2, respectively.

Compared to TgcS *A. baumannii* genome sequences (an unpublished lab resource), TgcR contained more ARG elements, especially *aph6id*, *aph3ib*, and *teta*, which encode aminoglycoside O-phosphotransferase, and major facilitator superfamily transporter tetracycline efflux pump, respectively (Supplementary Figure S1). Compared to 26 *A. baumannii* strains retrieved from GeneBank and used for phylogenetic analysis (Supplementary Figure S2), the 19 TgcR strains in this study lacked the *Streptomyces cinnamoneus* EF-Tu mutant gene, which confers elfamycin resistance. Interestingly, strains BJAB07105, MDRZJ06, BJAB07104, BJAB0868, and XH386, isolated in East and Northeast China, all contained this gene, whereas the remaining ARG patterns were similar to our strains. Compared to geographically distant strains, our hospital-isolated TgcR strains adopted more ARGs (*tetA*, *bla*_OXA–__66_, *armA*, *mphG*, and *msrE).*

#### Virulence Genes Annotated Using Virulence Factors of Bacterial Pathogens (VFDB) Database

**FIGURE 5:**
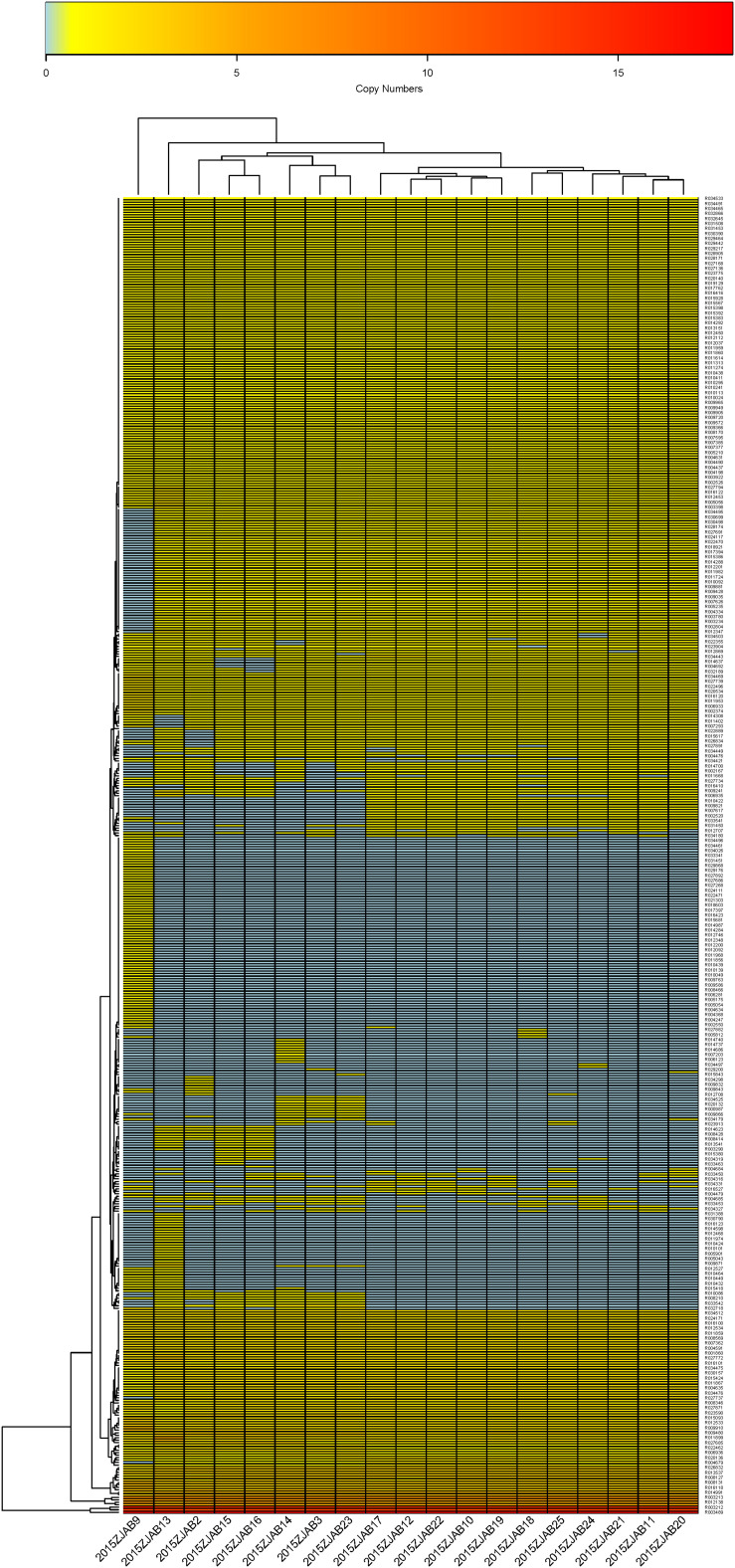
The heatmap of virulence gene number distribution in the 19 TgcR strains.

A total of 452 virulence genes were annotated by VFDB in all 19 strains, with 2015ZJAB13 having the most VFs (296) and 2015ZJAB23 having the least (263). In total, 391 VFs were found to be diversely distributed in all TgcR strains. The transcriptional regulator *Y* and *cylG* were the most annotated VF among all strains. Isolates from Patient 1 had 84 different VFs, whereas those from patients 2 and 3 had 59 and 9 different VFs, respectively. Based on the VF number distribution, 74 VFs were present with multiple VFs in all strains except 2015ZJAB9, whose VF profile was unique. The 19 TgcR strains can be divided into four sub-groups based on VF number distribution (Figure 5). VF patterns within each sub-group were similar, while marker genes were different between sub-groups. It should be noted that strains 2015ZJAB9 and 2015ZJAB13 had distinct VF profiles that were similar to the first two sub-groups regarding genes that were present in high numbers. These profiles resembled sub-group four with VFs distributed in low numbers. However, they possessed some unique VFs. The four sub-groups were separable using the VF markers indicated in Figure 5.

Major virulence factors in *A. baumannii* involved seven categories of elements (Adherence: OmpA; Biofilm formation: AdeFGH efflux pump, Bap, Csu fimbriae and PNAG; Enzymes: Phospholipase C and D; Immune evasion: Capsule and LPS; Iron uptake: Acinetobactin; Regulation: BfmRS and Quorum sensing and Serum resistance: PbpG). Although the VFs were diversely distributed among the 19 TgcR strains, the first two sub-groups and the latter two sub-groups could be differentiated using VFs R033541, R012040, R010421, R004310, R009821, R010419, R006935, R010422, R007617, R002520, and R007621, representing *cdiB*, *pvdN*, *wbpD*, *pilA*, *1sgF*, *wbpB*, *mip*, *wbpE*, *wbmB*, *cap8J*, and *BAV0081* genes, coding for hemolysin activator Hly, pvdN in *Pseudomonas_aeruginosa*, probable acetyltransferase WbpD, fimbrial protein, probable aminotransferase, putative UDP-galactose–lipooligosaccharide galactosyltransferase, probable oxidoreductase WpbB, macrophage infectivity potentiator (Mip), probable aminotransferase WbpE, wbmB hypothetical protein, capsular polysaccharide synthesis enzyme Cap8J, and putative O-antigen translocase, respectively. Compared to the 19 TgcR strains and geographically proximal isolates from Eastern and Northeast China, the remaining reference genomes lacked 36 VFs from R021300 to R030574 (Supplementary Figure S3), but possessed an additional 12 VFs from R027708 to R031413 (*lipP* acetyl-hydrolase/esterase, putative_acyl-CoA_dehydrogenase *fadD13*, *farB* antibacterial fatty acid resistance protein B, *senX3*, *fadD13* putative chain–fatty acid CoA ligase, *bprC* AraC family transcriptional regulator, *mgtB* ATPase, and *capD* gamma-glutamyltranspeptidase). Notably, BJAB0715 and 2015ZJAB9 were clustered with strains isolated from western countries, indicating a western origin and epidemical spread to China. ACICU, AC29, and AC30 from Rome and Malaysia were clustered with our strains and isolates from East and Northeast China and Taiwan, indicating their close evolutionary relationship.

#### KEGG Pathway Analysis and GO and COG Classification

GO terms of the 20 strains were annotated. In all, 39 GO terms were annotated: 26 in Group 1 (Patient 1), 23 in Group 2, and 21 in Group 3. COG categories were analyzed and compared.

KEGG pathway metabolic characteristics of the strains were analyzed. Comparatively, KEGG pathways varied the most in Group 1, and the most variable pathways were Replication and repair, Drug resistance, Metabolism of cofactors and vitamins, and Nucleotide metabolism.

## Discussion

Sequence types analysis of the 90 TgcR *A. baumannii* strains (tested by disk diffusion) collected during the first 18 months of Tgc usage in the hospital revealed that the most prevalent ST was ST195 (57.8%), followed by ST208, ST191, and ST784. In comparison to traditional strain typing approaches, such as PCR and sequencing-based methods targeting a few conserved genes, genome-wide comparative analysis provides a comprehensive and SNP-level resolution. Currently, the gene *gpi* is the most efficient and widely used locus for typing *A. baumannii* clinical strains.

When strains isolated from the same patient during antibiotic treatment were grouped together, a comparison within each group revealed their intricate genomic relationship and elucidated possible gene transfer events (21). For example, the two strains obtained from Patient 1 (2015ZJAB12 and 2015ZJAB13) had a limited number of SNPs (1,378) associated with different STs, indicating exchange of genomic sequences that possibly contained the conserved ST *gpi* gene. The two strains derived from Patient 2 had highly similar genomes. In comparison, the strains from Patient 3 were vastly different, suggesting that they might stem from separate infections.

Most of the TgcR *A. baumannii* strains were isolated from sputum, drainage fluid and blood, which were obtained from ICU, sICU, and Department of Internal Medicine settings (Supplementary Table S2). All strains were resistant to at least three categories of antibiotics (Table 1). When examining the patients’ ward and hospital stay times, there was no apparent overlap between site or time of isolation and genomic similarity, indicating spontaneous occurrence of resistance.

Mobile elements including GIs, ISs, and integrons were annotated. A total of 82 kinds of ISs belonging to 13 families were identified in 20 TgsS strains. IS*Vsa3* and IS*Aba26* were the most prevalent IS types, followed by IS*Aba1*, IS*Aba21*, IS*Aba33*, IS*26*, IS*15DI*, IS*15DII*, and IS*15DIV*, which were present in 18 out of 20 strains. The least common IS types were IS*Aha1*, IS*Aha2*, IS*Aba7*, IS*Aba12*, IS*Aba13*, IS*17*, and IS*Alw1*, which were only present in a specific strain, 2015ZJAB13. Multiple AR genes were found in this mobile element. Strain 2015ZJAB13 had the highest percentage of repeated sequences, which was in accordance with most ISs, suggesting an active genetic acquisition history.

In total, 24 ARGs (*bla*_OXA–__66_, *gyrA*, *adeR*, *adeS*, *mphG*, *armA*, *msrE*, *adeN*, *bla*_OXA–__23_, *lrfA*, *bla*_TEM–__1_, *aph3ia*, *bla*_OXA–__435_, *catB8*, *aadA*, *sul1*, *bla*_TEM–__122_, *bla*_CTX–M–__55_, *gyrB*, *EF-Tu mutant*, *bla*_OXA–__316_, *mel*, *sul2*, *and adeC)*, annotated by CARD, were present with different ARG number distribution patterns in the 19 TgcR isolates. This provided a genotyping method without consideration of SNPs. The 19 TgcR strains can, thus, be divided into three sub-groups (Figure 4 and Supplementary Figure S2). Strains within each sub-group shared similar ARG number distribution patterns, whereas different sub-groups were discriminated by a few marker genes. ARGs and their gene number diversity in order of prevalence were *lrfA* and *adeC* (19/19) (present in all strains including 2015ZJAB9 with different gene numbers), *bla*_OXA–__66_ (1/19) (absent only in 2015ZJAB9), *adeR* (18/19), *gyrA* (17/19), *adeS* (16/19), *mphG* (15/19), *armA* (15/19), *adeN* (15/19), *APH3’1a* (10/19), *bla*_OXA–__435_ (10/19), *sul1* (5/19), *catb8* (4/19), *aadA* (3/19), and EF-Tu mutant (2/19) (present only in strains 2015ZJAB9 and 2015ZJAB13), *mel* (2/19) (present only in strains 2015ZJAB21 and 2015ZJAB22) and *bla*_TEM–__122_, and *bla*_CTX–M–__55_ found only in 2015ZJAB17. In the grouping analysis, sub-group 1 was characterized by the presence of *bla*_OXA–__23_ and absence of *bla*_OXA–__316_. ARGs *mel*, *bla*_TEM–__122_, *bla*_CTX–M–__55_, and *bla*_OXA–__23_ were only present in the first sub-group strains. *Sul2* was a differentiating gene present in about half of sub-group 1 strains that appeared only once in sub-group 2. The unique and most abundant ARGs carried by the third sub-group were *catB8*, *aadA*, *gryB*, and *EF-Tu*. A lack of *mphG* and *armA* was a marker of the second sub-group.

Strain 2015ZJAB9 had the most unique ARG pattern. Comparison to genomes retrieved from GeneBank revealed its close phylogenetic relationship with BJAB0715, ZW85-1, LAC-4, AB031, D1279779, and D36, which were isolated in China, United States, Canada, and Australia, respectively. Strain 2015ZJAB9 shared similar ARG patterns with those isolated from France, America, Canada, and Australia, such as AYE, A1, AB030, and AB0057. These strains harbored a *S. cinnamoneus* EF-Tu mutant gene responsible for resistance to elfamycin, which is a main feed supplement in animal husbandry in western countries. Conversely, this EF-Tu mutant gene was absent in the TgcR strains in this study and in AC29, AC30, and ACICU isolates from Malaysia and Italy. Interestingly, MDR-ZJ06, MDR-TJ, BJAB07104, BJAB0868, XH386, TYTH-1, and TCDC-AB0715, which were isolated in East and Northeast China and Taiwan, contained this EF-Tu gene, while the remaining ARG profiles in these strains were more similar to those of strains isolated in neighboring regions, indicating a connection between geographic vicinity and acquisition of the gene.

Compared to ARGs of TgcS *A. baumannii* genomes, TgcR strains contained more ARGs including *aph6id*, *aph3ib*, and *teta*, which encode aminoglycoside O-phosphotransferase and major facilitator superfamily transporter tetracycline efflux pump, respectively (Supplementary Figure S1). As shown in Supplementary Figure S2, ARGs in the TgcR strains in this study were more similar to MDR-ZJ06, MDR-TJ, BJAB07104, BJAB0868, XH386, TYTH-1, and TCDC-AB0715 lacking the EF-Tu-encoding gene and having a greater abundance of *msrE*, *mphG*, *armA*, and *Propionibacterium acnes gyrA* than to MDR *A. baumannii* genomes retrieved from GeneBank. Among all strains that were isolated in East China, those from this study had the highest presence of *tetA*, *OXA-66*, *armA*, *mphG*, and *msrE* genes.

Using the same methodology, we analyzed VF features of TgcR strains. In total, 371 VFs were annotated in TgcR strains, with sample 2015ZJAB13 having the most (221 VFs) and 2015ZJAB23 having the least (188 VFs). The transcriptional regulator *cpsY* was the most annotated VF in all strains. *CylG* also had a higher ratio of annotation. Based on VF gene number diversity, TgcR strains can be divided into four sub-groups. VFs (R033541, R012040, R010421, R004310, R009821, R010419, R006935, R010422, R007617, R002520, and R007621) can differentiate between the first two sub-groups and the last two sub-groups. Compared to the TgcR strains and geographically proximal isolates from East and Northeast China, the remaining reference genomes lacked 36 VFs from R021300 to R030574 (Supplementary Figure S3), but possessed an additional 12 VFs from R027708 to R031413. Notably, BJAB0715 and 2015ZJAB9 were clustered with strains isolated from western countries, indicating a western origin and epidemical spread to China. While ACICU, AC29, and AC30 from Rome and Malaysia were clustered with our strains and isolates from East and Northeast China and Taiwan, indicating their evolutionally close relationship.

Taken together, ARGs and VFs may have coordinated interactions and interplay in survival and environmental adaptation. Metabolic activities and pathways in three strains under different concentrations of tigecycline were analyzed, and the differential transcript expression profiles suggested relationship with Tgc resistance. Further transcriptome analysis in all TgcR strains may provide more comprehensive information about gene regulation under antibiotic stress.

In summary, this study provides a survey of clinical TgcR *A. baumannii* isolates in a tertiary hospital in Eastern China during the first 2 years of adoption of Tgc in clinical use. We analyzed important functional ARG and VF elements, highlighting a dynamic genomic background in 19 TgcR *A. baumannii* strains. Herein, we demonstrate the possibility of using a simple approach of ARG number distribution analysis to sub-group TgcR *A. baumannii*. This facilitates the resistome profile characterization and comparison. The distinct yet traceable occurrence of TgcR *A. baumannii* has raised concern for medical workers. Genome plasticity revealed by our study provided a basis for the development of Tgc resistance, especially under environment pressure such as antibiotic treatment. Possible mechanisms include frequent genetic material exchange, homologous recombination of large genomic fragments, re-infection of resistant bacteria, changes in transcriptional regulation of efflux pumps, or induction by other antibiotics. Elucidation of these mechanisms requires further investigation.

## Data Availability Statement

The datasets generated for this study can be found in the NCBI with accession numbers PRJNA593931 and PRNJNA307212.

## Author Contributions

LL and YX designed the study. LL managed the project, analyzed the data, and wrote the manuscript. LL, PS, BZ, WY, and JJ collected the samples and performed the bacterial antibiotic susceptibility tests. LL and PS preformed the DNA extraction, library construction, and sequencing experiments. YX revised the manuscript. All authors contributed to the article and approved the submitted version.

## Conflict of Interest

The authors declare that the research was conducted in the absence of any commercial or financial relationships that could be construed as a potential conflict of interest.

## Footnotes

1 www-is.biotoul.fr

2 ftp://ftp.ncbi.nlm.nih.gov/genomes/genbank/bacteria/

## Supplementary Material

FIGURE S1 The heatmap of the ARG number distribution in the tigecycline-resistant (TgcR) and tigecycline-sensitive (TgcS) strains. The 2015ZJAB strains that were tigecycline resistant are represented by triangles, and SN strains that were tigecycline sensitive are represented by diamonds.

FIGURE S2 The heatmap of the ARG number distribution in the TgcR and *Acinetobacter baumannii* strains retrieved from GeneBank. Tigecycline-resistant isolates are represented by triangles, tigecycline-sensitive isolates are represented by stars, and isolates with unknown tigecycline susceptibility are represented by diamonds.

FIGURE S3 Comparison of VF number distribution in the 19TgcR strains and geographically close isolates from Eastern and Northeast China.

TABLE S1 The MLST data of 90 clinically isolated *Acinetobacter baumannii* strains.

TABLE S2 Clinical information of 56 TgcNS strains isolated from 36 patients.

TABLE S3 General features of 19 TcgR *A. baumannii* genomes.
