# Supplementary figures and images for: Comparative Genomic Analysis of 19 Clinical Isolates of Tigecycline-Resistant Acinetobacter baumannii
Source: Front Microbiol. 2020 Jul 7;11:1321. doi: 10.3389/fmicb.2020.01321 (PMC7358374; doi:10.3389/fmicb.2020.01321)

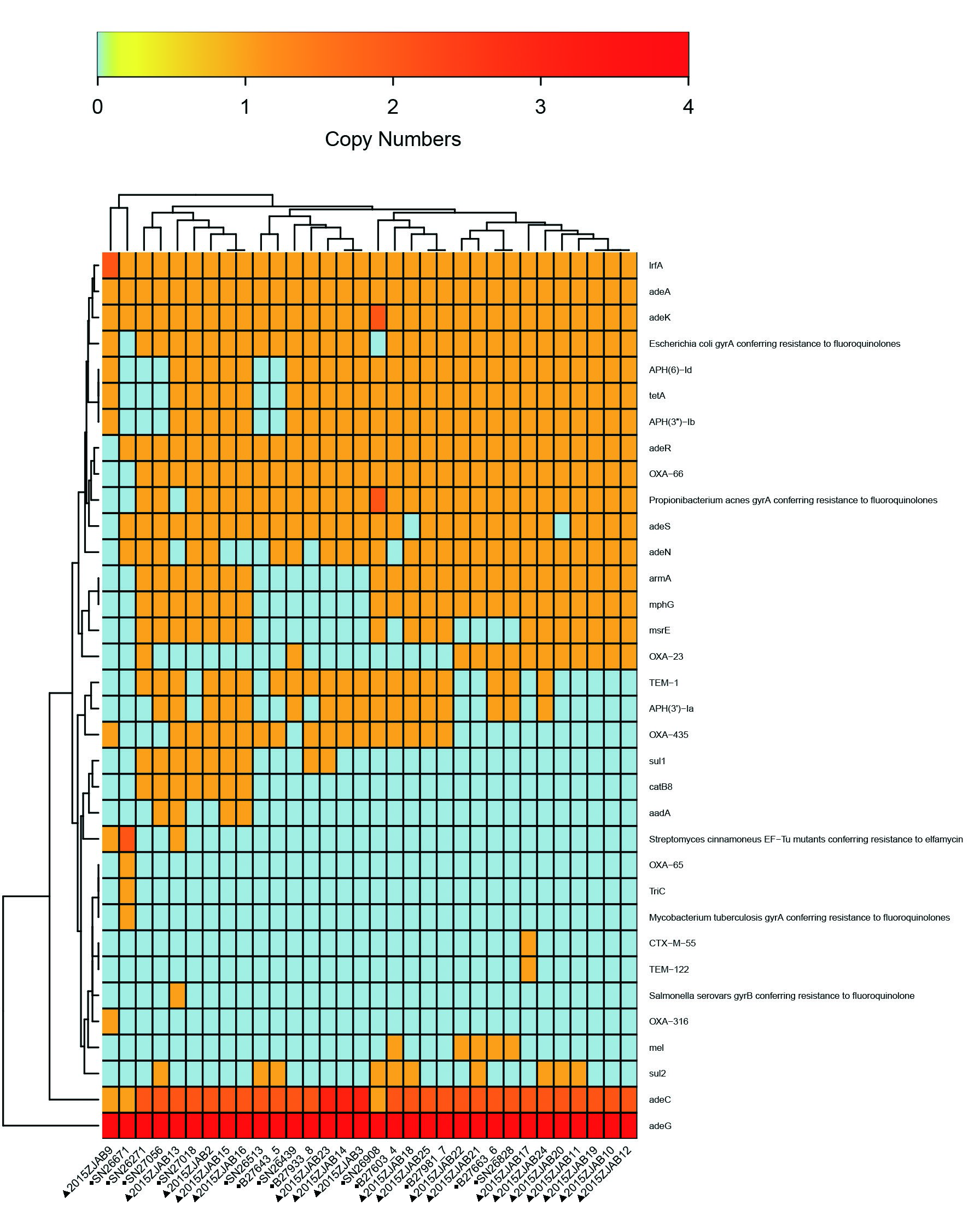

Supplement: FIGURE S1 — The heatmap of the ARG number distribution in the tigecycline-resistant (TgcR) and tigecycline-sensitive (TgcS) strains. The 2015ZJAB strains that were tigecycline resistant are represented by triangles, and SN strains that were tigecycline sensitive are represented by diamonds. [file Image_1.TIFF]

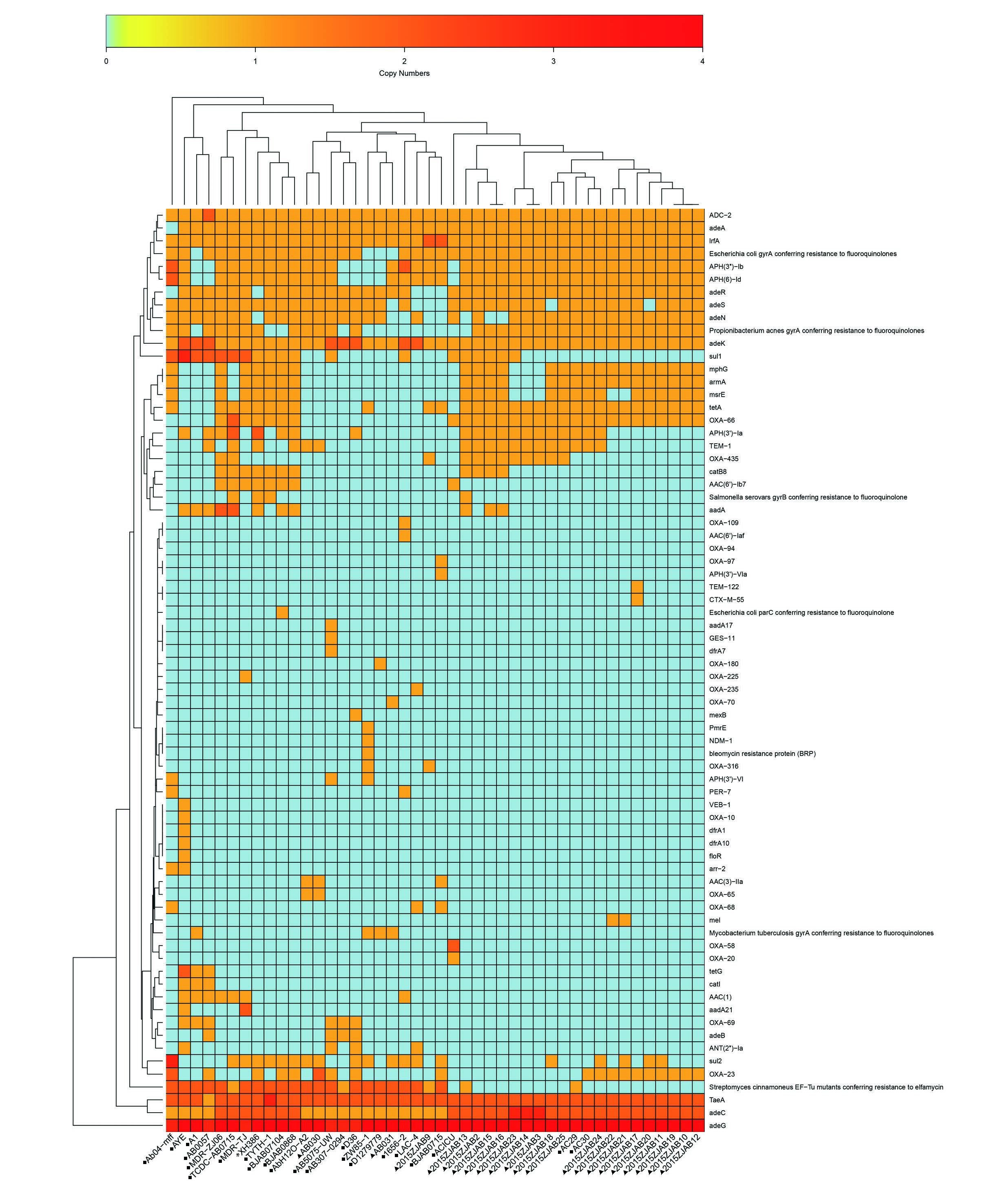

Supplement: FIGURE S2 — The heatmap of the ARG number distribution in the TgcR and Acinetobacter baumannii strains retrieved from GeneBank. Tigecycline-resistant isolates are represented by triangles, tigecycline-sensitive isolates are represented by stars, and isolates with unknown tigecycline susceptibility are represented by diamonds. [file Image_2.TIFF]

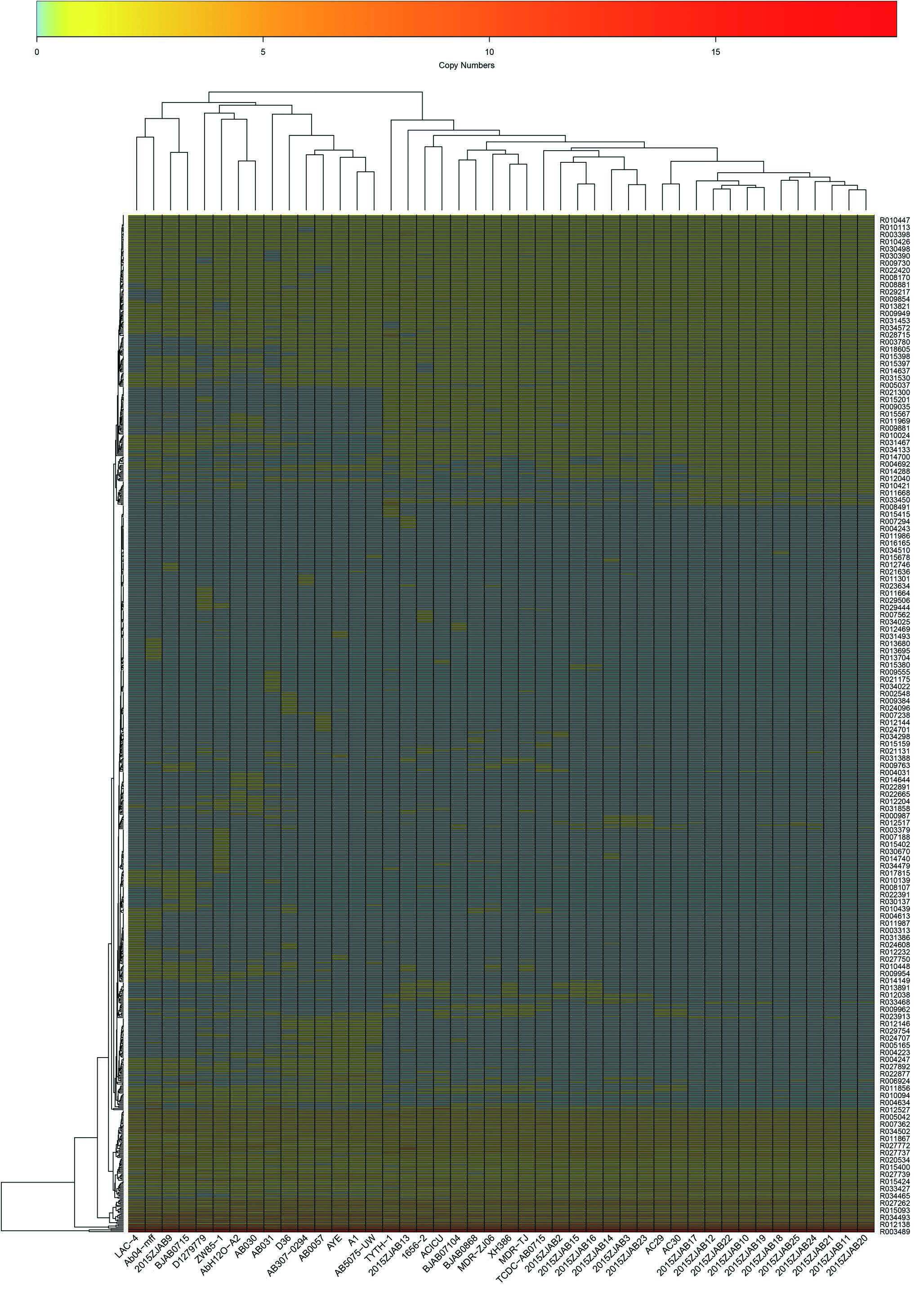

Supplement: FIGURE S3 — Comparison of VF number distribution in the 19TgcR strains and geographically close isolates from Eastern and Northeast China. [file Image_3.tiff]
