# Supplementary material for: Comparative Genomic Analysis of 19 Clinical Isolates of Tigecycline-Resistant Acinetobacter baumannii
Source: Front Microbiol. 2020 Jul 7;11:1321. doi: 10.3389/fmicb.2020.01321 (PMC7358374; doi:10.3389/fmicb.2020.01321)
Supplement: TABLE S1 — The MLST data of 90 clinically isolated Acinetobacter baumannii strains. [file Table_1.DOCX]

**Supplementary Table 1. The MLST data of 90 clinically isolated *A. baumannii* strains.**

| ST-Oxford | Loci | No. | Ratio (%) |
| --- | --- | --- | --- |
| 195 | 1-3-3-2-2-96-3 | 52 | 57.8 |
| 208 | 1-3-3-2-2-97-3 | 8 | 8.9 |
| 191 | 1-3-3-2-2-94-3 | 6 | 6.7 |
| 784 | 1-3-3-2-2-107-3 | 6 | 6.7 |
| 214 | 1-3-3-2-2-96-2 | 5 | 5.6 |
| 642 | 22-15-13-12-4-169-2 | 3 | 3.3 |
| 643 | 1-3-3-2-2-169-3 | 3 | 3.3 |
| 451 | 1-3-3-2-2-142-3 | 3 | 3.3 |
| 547 | 1-3-3-2-2-157-3 | 2 | 2.2 |
| 368 | 1-3-3-2-2-140-3 | 1 | 1.1 |
| 369 | 1-3-3-2-2-106-3 | 1 | 1.1 |

The seven conserved loci were listed in the order of *gltA*, *gyrB*, *gdhB*, *recA*, *cpn60*, *gpi* and *rpoD.*
