# Supplementary material for: Comparative Genomic Analysis of 19 Clinical Isolates of Tigecycline-Resistant Acinetobacter baumannii
Source: Front Microbiol. 2020 Jul 7;11:1321. doi: 10.3389/fmicb.2020.01321 (PMC7358374; doi:10.3389/fmicb.2020.01321)
Supplement: TABLE S2 — Clinical information of 56 TgcNS strains isolated from 36 patients. [file Table_2.DOCX]

**Supplementary Table 2. Clinical information of 56 TgcNS strains isolated from 36 patients.**

|  | | TgcNS | TgcS | *P* value |
| --- | --- | --- | --- | --- |
| Male/Female(no.) | | 25/11 | 14/6 | 0.965 |
| Age (±SD） |  | 57.1±18.05 | 59.3±14.68 | 0.848 |
| Wards | ICU | 13 | 10 | 0.548 |
|  | sICU* | 8 | 4 | 0.846 |
|  | Internal Medicine | 8 | 4 | 0.846 |
|  | Other | 7 | 2 | 0.356 |
| Stay duration  （d）(±SD) | Before Isolation | 15.61±11.64 | 17.5±15.92 | 0.530 |
|  | Total duration | 44.2±26.7 | 49.56±49.35 | 0.145 |
| Sample types | Sputum | 33 | 14 | 0.022 |
|  | Drain fluid | 10 | 2 | 0.202 |
|  | Blood | 5 | 1 | 0.473 |
|  | Cather culture fluid | 5 | 2 | 0.947 |
|  | Bile | 2 | 1 | 0.640 |
|  | Ascetic fluid | 1 | 0 | 0.463 |
| Antibiotic usage | Piperacillin- Tazobactam | 16 | 1 | 0.002 |
|  | Carbapenem | 23 | 11 | 0.514 |
|  | 3-Cephalosporin* | 6 | 3 | 0.871 |
|  | Quinolones | 17 | 1 | 0.001 |
|  | Cefoperazone  Sulbactam | 22 | 5 | 0.010 |
|  | tigecycline | 9 | 0 | 0.015 |

Note: This table clinical information of 56 TgcNS (19 TgcR and 37 TgcI) *A. baumannii* strains and 36 patients where they were isolated. *3-Cephalosporin stand for the third generation cephalosporins. Also clinical information of 20 randomly-selected TgcS strains and 20 patients where they were isolated were also included for comparison.
