# Supplementary material for: Comparative Genomic Analysis of 19 Clinical Isolates of Tigecycline-Resistant Acinetobacter baumannii
Source: Front Microbiol. 2020 Jul 7;11:1321. doi: 10.3389/fmicb.2020.01321 (PMC7358374; doi:10.3389/fmicb.2020.01321)
Supplement: TABLE S3 — General features of 19 TcgR A. baumannii genomes. [file Table_3.DOCX]

**Supplementary Table 3. General features of 19 TcgR *A. baumannii* genomes.**

|  | **2015ZJAB2** | **2015ZJAB3** | **2015ZJAB9** | **2015ZJAB10** | **2015ZJAB11** | **2015ZJAB12** | **2015ZJAB13** | **2015ZJAB14** | **2015ZJAB15** | **2015ZJAB16** |
| --- | --- | --- | --- | --- | --- | --- | --- | --- | --- | --- |
| **Sample type** | sp | sp | sp | dr | ca | sp | sp | ca | sp | sp |
| **Q30 (%)** | 94.74 | 93.87 | 89.40 | 88.95 | 89.00 | 89.80 | 90.11 | 89.96 | 89.86 | 89.52 |
| **Clean data (Mbp)** | 671 | 668 | 649.5 | 612 | 631.5 | 612 | 654 | 612 | 654 | 612 |
| **Coverage (%)** | 95.99 | 95.88 | 87.44 | 98.08 | 98.09 | 98.11 | 92.77 | 95.85 | 96.17 | 96.21 |
| **GC (%)** | 38.92 | 38.99 | 38.81 | 38.81 | 38.83 | 38.82 | 38.86 | 38.85 | 38.97 | 38.98 |
| **Scaffold num** | 53 | 41 | 64 | 50 | 48 | 47 | 163 | 37 | 75 | 74 |
| **Scaffold N50 (bp)** | 177,950 | 177,986 | 129,831 | 194,221 | 167,876 | 194,221 | 133,516 | 236,720 | 149,403 | 155,965 |
| **Scaffold N90 (bp)** | 50,405 | 72,813 | 37,032 | 48,417 | 50,614 | 48,147 | 23,520 | 73,936 | 43,957 | 54,630 |
| **Contig num** | 58 | 45 | 111 | 84 | 84 | 81 | 382 | 62 | 129 | 126 |
| **CDS num** | 3,753 | 3,708 | 3,817 | 3,725 | 3,712 | 3,713 | 4,090 | ,3781 | 3,800 | 3,792 |
|  | **2015ZJAB17** | **2015ZJAB18** | **2015ZJAB19** | **2015ZJAB20** | **2015ZJAB21** | **2015ZJAB22** | **2015ZJAB23** | **2015ZJAB24** | **2015ZJAB25** |  |
| **Sample type** | dr | sp | sp | sp | sp | bi | bi | sp | bi |  |
| **Q30 (%)** | 89.31 | 89.00 | 89.80 | 90.38 | 89.36 | 89.01 | 88.73 | 89.48 | 89.60 |  |
| **Clean data (Mbp)** | 612 | 612 | 612 | 631.5 | 592.5 | 612 | 592.5 | 612 | 612 |  |
| **Coverage (%)** | 97.96 | 98.18 | 98.08 | 98.1 | 98.09 | 98.08 | 95.89 | 98.17 | 98.17 |  |
| **GC (%)** | 38.84 | 38.85 | 38.82 | 38.81 | 38.82 | 38.83 | 38.88 | 38.83 | 38.83 |  |
| **Scaffold num** | 54 | 52 | 51 | 50 | 51 | 50 | 40 | 53 | 52 |  |
| **Scaffold N50 (bp)** | 184,674 | 184,675 | 130,493 | 167,876 | 133,307 | 184,696 | 175,147 | 167,876 | 167,876 |  |
| **Scaffold N90 (bp)** | 50,710 | 48,147 | 65,489 | 50,765 | 51,059 | 48,147 | 70,496 | 43,178 | 48,412 |  |
| **Contig num** | 95 | 80 | 99 | 94 | 91 | 98 | 67 | 88 | 89 |  |
| **CDS num** | 3,729 | 3,711 | 3,713 | 3,729 | 3,723 | 3,713 | 3,795 | 3,722 | 3,726 |  |
